# Hearing improvement among children with cholesteatoma who underwent canal wall up and canal wall down surgical management

**DOI:** 10.12669/pjms.38.4.4933

**Published:** 2022

**Authors:** Waseem Ahmad, Sehrish Akbar, Shazia Hassan

**Affiliations:** 1 Dr. Waseem Ahmad, MBBS, MO, Department of ENT, Nishtar Medical University & Hospital, Multan, Pakistan; 2 Dr. Sehrish Akbar, MBBS, WMO, Department of ENT, Nishtar Medical University & Hospital, Multan, Pakistan; 3 Dr. Shazia Hassan, MBBS, WMO, Department of ENT, Nishtar Medical University & Hospital, Multan, Pakistan

**Keywords:** Cholesteatoma, Canal wall up, Canal wall down, Surgical management, Hearing improvement

## Abstract

**Objectives:**

The most frequently used surgical methods for treating cholesteatoma include canal wall up and canal wall down procedures. The objective of the study was to compare the hearing improvement among children with cholesteatoma who underwent canal wall up and canal wall down surgical management.

**Methods:**

The cross-sectional analytical study design was used. The study was conducted at the ENT Department of Nishtar Medical University & Hospital Multan from 15^th^ June to 15^th^ Nov 2020.. Forty six patients with cholesteatoma were enrolled in the study after taking informed consent. Inclusion and exclusion criteria were followed. The participants were categorized into two groups. Group-A was treated with canal wall-up surgery while Group-B was treated with canal wall down Mastoidectomy. A 12-month post-operative follow-up and the audiometry assessment were compared with pre-surgical values. Additionally, a COMOT-15 survey was administered to analyze self-perceived hearing functions. The Chi-square test was used for comparative analysis of the surgical outcome and hearing improvement among the two groups. P-value (p value<0.05) was considered statistically significant.

**Results:**

Forty six patients were included in the study with 23 participants in each group. Among 46, 26 were male and 20 were female. The pre and post-operative mean Pure-tone average values were significantly different in (Group-A) who underwent canal wall up Mastoidectomy (p<0.05) than in Group-B, who underwent canal wall down Mastoidectomy. Similarly, hearing sub-section responses of the COMOT-15 survey favored the Canal wall technique. However, the survey showed no significant differences in the mental health status of the two groups (p<0.05).

**Conclusion:**

Our data collected after a one-year follow-up of patients suggests canal wall up as a preferred technique for hearing improvement than canal wall down technique.

## INTRODUCTION

Cholesteatomas are characterized as congenital or acquired destructive growth of the stratified squamous epithelium in the temporal bone.^1^ Due to progressive expansion of the keratinous material, serious complications are reported as the surrounding structures get eroded.^2^ In every 100,000 inhabitants, 1-12 cases of cholesteatomas are reputed worldwide.^3^ Although the clinical manifestation of the disease may vary between children and adults, patients usually experience constant or periodic fetid and aural discharge whereas complaint of pain is unusual and the extent of hearing loss varies from patient to patient. In critical cases, the facial nerve, auditory system, and vestibular system get involved which might cause sensorineural or mixed hearing loss, vertigo, facial palsy, or tinnitus.

Given the severity of associated consequences, surgery of cholesteatoma aim not only to get rid of the bone or mucosal disease but also to ensure that recurrence of the disease is prevented. However, surgeries did not always meet their goals and either cholesteatoma may persist (residual disease) due to failure to eliminate or a recurrent cholesteatoma appears that is mostly secondary to the retraction pocket. Therefore, researchers are still investigating different surgical techniques to identify a preferable efficient surgical procedure.^4^

### Mastoidectomies are of two types:

canal wall up (CWU) and canal wall down (CWD). In the latter technique, a cavity or mastoid bowl is created which usually fills up with earwax and requires frequent cleaning of the ear canal and water protection. Besides, in the CWD surgery architecture of the ear is slightly changed which might disturb the hearing ability to certain degrees.^5^ Therefore, CWU mastoidectomy was introduced to counter the limitations of CWD mastoidectomy but later on, it was found that it results in a higher recurrence rate.^6^ Thus, recent researches have been focusing to evaluate the outcomes of surgeries not only in terms of their technical success but also in regards to their contribution to improving the lifestyle of the patient.^4^ The rationale of the current study is based on the belief of the author to validate a surgical strategy with the better, long-term effect of hearing ability of the patients. The objective of the study was to compare the Hearing improvement among children with cholesteatoma who underwent canal wall up and canal wall down surgical management.

## METHODS

A cross-sectional analytical study was conducted in the ENT Department of Nishtar Medical University & Hospital Multan from 15^th^ June to 15^th^ Nov 2020. A total of 46 patients, who were non-responsive to repeated cycles of medical therapy, were reporting the gradual hearing loss and thus were candidates for surgery, were enrolled in the study through consecutive sampling technique. The sample size was calculated 80% value of the power set and p-value less than 0.05. HR CT scan of the temporal bone was done to confirm the disease which showed inflammation in the middle ear along with slight erosion of the associated bone structures. Patients who matched the following criteria were excluded from the study: patients older than 13 years, those with bilateral disease and were guided to undergo variable surgical techniques in both ears, those who were having their revision surgery, the patients who had planned tympanoplasty without Mastoidectomy, and those with other comorbidities which might influence their quality of life and in turn produce bias in the study results were excluded.

After the informed consent of the patients and ethical approval ref#36/179 dated 01/06/20 from the hospital, the participants were divided into two groups. Group-A comprised of 23 patients who had extensive middle ear growth and erosion of the external ear canal, therefore underwent CWD technique (CWD group). Whereas Group-B comprised of other 23 patients who reported limited disease progress, therefore CWU tympanomastoidectomy was performed on them (CWU). Standard protocols were followed during surgery on participants of both the groups and a retro auricular incision with a tympanometry flap was made in all the cases. Pure-tone audiometry (PTA) was performed on all the patients for testing the traditional frequency range (0.24 to 8 kHz) in a soundproof room. Pure-tone average (PTA) values were measured as the mean of 500, 1000, 2000, and 4000Hz thresholds. The audio logical assessment was performed 24 hrs. Before the surgery and was later compared with 12 months following the surgery. In addition to post-operative audio logical testing, patients were also administered Chronic Otitis Media Outcome Test-15 (COMOT-15) survey. The survey is composed of fifteen items and three subscales hearing function (HF), mental health (MH), and ear symptoms (ES).

### Statistical analysis:

Version 20.0 was used for all the statistical analyses. Continuous variables were presented in the form of mean ± standard deviation (SD). The Chi-square test was used to compare the outcomes between the two groups. P values< 0.05 were considered statistically significant for any variable.

## RESULTS

A total of 46 patients were enrolled in the study with a mean age of 6.7 ± 5.1 years and 26 males and 20 females. In the CWD group, the mean age of the patients was 7.2 (SD = ± 3.5). In the CWU group, 8.1 (SD = ± 4.4) was the mean age of the patients. All the patients were followed for a mean time of 12 months (SD = ± 2). No recurrent or residual cholesteatoma was reported at the time of data collection following the surgery.

The pre-operative mean PTA of all the participants was 48 dB (range = 12-95; SD = ± 20). In the CWD group, the patients had pre-operative PTA value of 51 dB (range = 16-95; SD = ± 19), while in the CWU group, mean PTA was 43 dB (range = 9-95; SD = ± 25). The two groups differ significantly (p<0.05) in terms of pre-operative PTA value. 12-month post-operative assessment noted that overall mean PTA for all the patients was 45 dB (range = 10-90; SD = ± 19). In the CWD group, the mean post-surgical PTA was 49 dB (range = 20-79; SD = ± 16), while in the CWU group the average post-surgical PTA was 40 dB (range = 15-68; SD = ± 17). The mean postoperative PTA values of the two studied groups differ significantly (p<0.05). The intraGroup-Analysis found no significant difference in the pre-operative and post-operative PTA values. Ppre-and post-operative audiological data of both the studied groups. Is shown in Table-I. Inter-group comparison of COMOT-15 scores showed no significant difference in terms of mental health & air bone gap reduced while hearing function score significantly favored the CWU group.

**Table-I:** Assessment of pre and post-surgical PTA data of both study groups (N=46).

		500 Hz	1000 Hz	2000 Hz	4000 Hz	Mean PTA	P-value
CWD	Pre-surgical PTA (dB) ± SD	54± 19	59 ± 18	53± 22	57± 20	51±19	p>0.05
	Post-surgical PTA (dB) ± SD	51± 18	53 ± 19	47± 20	52 ± 18	49± 16	
	Delta PTA (dB) ± SD	3± 18	6± 19	6± 17	4± 18	2± 18	
CHU	Pre-surgical PTA (dB) ± SD	49±18	51± 17	42±20	47±18	43± 25	p>0.05
	Post-surgical PTA (dB) ± SD	45±18	47±19	37±17	41±20	40±17	
	Delta PTA (dB) ± SD	4±16	4±17	5±20	6±18	3± 18	

## DISCUSSION

The study was aimed to compare widely employed surgical techniques in terms of their capacity to improve hearing in patients of cholesteatomata. The results found that CWU significantly improved the hearing ability of the patients when compared with the CWD technique; however, no significant improvement in the intraGroup-Audiometry results was observed pre- and post-operatively whereas COMOT-15 favored canal up Mastoidectomy as a preferable technique.

A similar study was conducted by Bhatt et al., who compared 6 and 12-week postoperative audiometry results between the two groups. The analysis showed that majority of the patients in both groups significantly improved; however, the patients who underwent the CWU technique showed better results as compared to the CWD technique.^7^ Similarly, Tos et al., observed that the hearing threshold became worse after canal wall down mastoidectomy, hence the study suggested adopting the CWU technique.^8^

Bhat S. et al conducted the study to compare hearing gain in the canal wall down vs canal wall up mastoidectomy. It was found that CWU had improved hearing better (18.36 dB) than that of the CWD technique.^9^ In another comparative study, Kalita S et al. observed that 3.3% of patients had an air-born gap (ABG) less than 30 dB in the CWD group while 6.67% in Intact Canal Wall (IWC) mastoidectomy. This ABG remained the same in 3.3% of patients three months post-operatively while it was observed in 20% of the IWC group. The results suggested that the IWC technique was preferable to CWD for shifting ABG of patients towards a better hearing range.^10^

In our study, the results of the audio logical assessment were overlapped with the outcomes of COMOT-15. The “hearing function” subsection validated the CWU technique over CWD. However, no significant difference was found in terms of mental health status. The existing studies based on COMOT-15 have established that diminished auditory function of patients is the most reported disabling symptom by the patients who undergo CWD as compared to CWU.^11^ This complies with the poorer functional data obtained by the survey of our study.

COMOT-15 assessment enables us to reveal a significant difference between the two study groups regarding their self-perceived hearing non-functionality. The literature indicates that hearing disability can be demonstrated along with other negative consequences, such as pain, drainage, and smell, through the administration of the Chronic Ear Survey questionnaire (CES).^12^ The ease of administration and evaluation of mental health, which is unavailable in CES, has made COMOT-15 a useful assessment tool for people with chronic hearing issues.

**Table-II:** COMOT-15 scores of the two studied groups.

	MH	HF
CWD (mean ± SD)	73±13	42±18
CHU	73±16	63±17
p-value	P>0.05	P<0.05

The correlation of results of audio logical testing and COMOT-15 survey has controversial interpretations in different studies. Similar to our study findings, Baumann et al. found that association only between COMOT-15 MH and HF subscales and hearing threshold.^13^ Whereas, Lailach et al. indicated the moderate relationship between COMOT-15 overall score and PTA and a strong association between the hearing function subscale and PTA.^14^ In consistence with our results, some studies have demonstrated partial or no correlation between questionnaire subsections and PTA. According to our data, the two analyzed groups don’t differ significantly in terms of psychiatric disturbances whereas Bakir and colleagues demonstrated a high incidence of mental disorders among the population affected by hearing impairment.^15^

### Limitations:

Our study is limited in terms of the classification of patients in two groups based on the extent of pathology. Since patients with the extensive disease were deliberately grouped to undergo the CWD technique, it might be possible that poorer hearing function results correlated with their extensive pathology as no significant intra-group improvement could be witnessed in any group. Similarly, patients were informed of the technique they underwent which might have produced bias in the survey results.

## CONCLUSION

Our data collected after a one-year follow-up of patients suggests canal wall up as a preferred technique for hearing improvement than canal wall down technique. However, long-term studies are required with a larger sample size and random placement of patients in two groups as we propose to address the limitations of this study in the future.

### Authors’ Contribution:

**WA, SA:** Conceived, designed and did statistical analysis & editing of manuscript.

**SA, SH:** Did data collection and manuscript writing.

**SH, WA:** Did review and final approval of manuscript.

**WA:** Accountable for the accuracy of the study.
